# Are Insect-Based Foods Healthy? An Evaluation of the Products Sold in European E-Commerce

**DOI:** 10.3390/foods14091450

**Published:** 2025-04-22

**Authors:** Emma Copelotti, Filippo Fratini, Giulia Sforza, Tiziano Tuccinardi, Gian Carlo Demontis, Simone Mancini

**Affiliations:** 1 Department of Veterinary Sciences, University of Pisa, Viale delle Piagge 2, 56124 Pisa, Italy; emma.copelotti@phd.unipi.it (E.C.); filippo.fratini@unipi.it (F.F.); g.sforza@studenti.unipi.it (G.S.); 2 Interdepartmental Research Center Nutrafood “Nutraceuticals and Food for Health”, University of Pisa, Via del Borghetto 80, 56124 Pisa, Italy; 3 Department of Pharmacy, University of Pisa, Via Bonanno 6, 56124 Pisa, Italy; tiziano.tuccinardi@unipi.it (T.T.); giancarlo.demontis@unipi.it (G.C.D.)

**Keywords:** entomophagy, nutritional value, edible insects

## Abstract

Over the past few years, edible insects have been recognised as potential “new” food sources in Western countries due to their sustainability and adaptability in the food production sector. To determine the distribution in Europe of insect-based food producers within each country, as well as the number and types of products, data from e-commerce were collected and analysed. The FoodEx2 classification was used to categorise the products. Data about the nutritional composition were recorded and the Recommended Daily Allowance (RDA) was calculated. As a result, 523 products offered by fifty-three companies located in 17 countries of Europe were found. The predominant market was based in Western Europe (55.8%), and 24 insect species were sold. Notably, four species were the most representative: *Tenebrio molitor* (182 products), followed by *Acheta domesticus* (140), *Alphitobius diaperinus* (54), and *Locusta migratoria* (34). Other species were present in lower quantities. The main commercial categories of insect-based food present in the European e-commerce were snacks, chocolate, and similar products. The results emphasise the potential benefits of incorporating insect-based food into the daily diet in terms of protein, energy, and fat intake. The RDA of the different products highlighted the importance of insects as a nutritional ingredient.

## 1. Introduction

One of the biggest problems of our time is to produce safe, nutritional, environmentally friendly, and affordable foodstuff. The rising global population, growing demand for protein food, escalating costs of animal–plant protein sources, food security concerns, and the environmental impact of intensive livestock and plant farming [1,2] lead to the growing interest in producing and consuming alternative protein sources all over the world. Over the last decade, the scientific community and producers have proposed several new food resources, such as micro and macro algae, single-cell proteins, and insects [3,4,5,6,7,8]. In this context, insect-based food represents an emerging and quite practical solution for the challenges that we are facing [9]. Entomophagy (the consumption of insects by humans) is an ancient dietary tradition still observed in various countries across Asia, Africa, and Latin America [10]. On the other hand, in Western countries, nowadays eating insects is related to the emotion of disgust [11], and it may appear as a threat to cultural identity since insects are not part of Western countries’ culinary traditions [12] or like a primitive or even barbaric practice [1]. Remarkably, due to sustainability and the potentiality and plasticity of the producing sector, in the last years edible insects were identified as potential “new” food sources in Western countries. Indeed, the intrinsic drivers related to the rearing of insects such as the low environmental impact and healthiness were the most important motivations to accept for Western people even though aversion and dislike were common motivations to reject insects [13]. In general, insects represent an excellent source of nutrients, particularly proteins and fats. The nutritional composition of insects varies between species and is also influenced by biotic and abiotic factors such as diet, rearing conditions, and the developmental stage [14]. In 2015, the European Food Safety Authority published its first scientific opinion on the risks of rearing and consuming insects as food and feed [15]. Edible insects and insect-based products in the European Union are classified as Novel Food, namely those products that humans had not consumed significantly in the EU before 15 May 1997 (Regulation (EU) 2015/2283, in force since 1 January 2018). Following Regulation (EU) 2015/2283, the commercialisation of Novel Food depends on the submission of an application by the company that is seeking to introduce that product to the market. The application must undergo an evaluation and authorisation process conducted by the European Commission (EC) and the European Food Safety Authority (EFSA). The EFSA is responsible for confirming the safety of a product for consumers [16,17,18]. Nine Novel Food applications received a positive opinion from EFSA so far for four different insect species. The last positive opinion by EFSA was released in January 2025 regarding the safety of frozen and dried forms of whole yellow mealworm (*Tenebrio molitor* larvae) (the European Commission Implementing Regulation is still pending). Looking back, the first authorised insect-based Novel Food product was the ‘dried’ *Tenebrio molitor* larvae (yellow mealworm). Indeed, on 13 January 2021, EFSA published a positive opinion, which became the European Commission Implementing Regulation (EU) 2021/882 on 1 June 2021. From 22 June 2021, the place on the EU market for the dried mealworm was authorised for the applicant and the associated business partners. Still, in 2021, the application for ‘dried and frozen’ *Locusta migratoria* (migratory locust) was authorised (Commission Implementing Regulation (EU) 2021/1975). In 2022, an application for ‘frozen and freeze-dried’ formulations of the yellow mealworm, ‘whole or in powder’, was authorised (Commission Implementing Regulation (EU) 2022/169). In the same year, the application for ‘dried, ground, and frozen’ *Acheta domesticus* (house cricket) was authorised (Commission Implementing Regulation (EU) 2022/188). In 2023, applications were authorised for the commercialisation of the ‘partially defatted powder’ forms of house cricket and *Alphitobius diaperinus* (lesser mealworm) ‘frozen, paste, dried, and powder’ formulations (Commission Implementing Regulation (EU) 2023/58). The last authorisation was released on 20 January 2025 authorising the placing on the market of UV-treated powder of whole *Tenebrio molitor* larvae (yellow mealworm) (Commission Implementing Regulation (EU) 2025/89). Applications are still pending dealing with the following species: *Gryllodes sigillatus*, *Hermetia illucens*, *Apis mellifera*, *Locusta migratoria*, *Tenebrio molitor*, *Alphitobius diaperinus*, and *Acheta domesticus*. Regarding extra-EU countries, in Switzerland, since 1 May 2017, the Federal Food Safety and Veterinary Office (FSVO) allowed the production and the market of three species of insects (*Tenebrio molitor*, *Acheta domesticus*, *and Locusta migratoria*) as food or ingredient in foodstuffs. All Novel Foods that can be marketed in the EU may be sold in Switzerland without authorisation. On the other hand, Swiss Novel Foods must be authorised by the European Commission to be placed on the EU market. Meanwhile, the legislative framework pertaining to edible insects in the United Kingdom attests that from the 1 January 2024 only products that have a valid Novel Food application presented before 31 December 2023 can be sold. Furthermore, the aforementioned products may only belong to the following four species: *Tenebrio molitor*, *Acheta domesticus*, *Gryllodes sigillatus*, and *Hermetia illucens*. In Norway, Novel Foods follow the Regulation 1215 of the 25 July 2017 which implements the Regulation (EU) 2015/2283, and the Norwegian Food Safety Authority is the competent organisation that food operators should consult for placing insect-based products on the market. Most of the companies selling insect-based products that are present in Europe are settled in France, the United Kingdom, Belgium, and the Netherlands [19]. As described by IPIFF’s survey [20], whole insects are the most commonly consumed form, followed by protein bars, snacks, and pasta. However, because of their absence in European food traditions, the insect sector still faces some difficulties related to many reasons such as the idea of a bad taste or even worse that it is too risky for health. Other motivations could be related to religion and diet limitations, difficulties in easily finding products in physical shops, and high prices [9,21,22]. Indeed, the predominant method of purchasing insect-based food products in the EU market is through e-commerce [23,24]. This study aims to conduct a thorough analysis of the current European market for insect-based food products considering insect species, price, percentage of insects included in the formulations, nutritional values, production country, and food category.

## 2. Materials and Methods

### 2.1. Search Procedure, Inclusion, and Exclusion Parameters

Data were collected online between July 2021 and January 2024. The research was performed on the Google web platform using different combinations of keywords: “(Edible insect* OR Novel food) AND (Europ* OR EU) AND (e-commerce OR shop online)” to identify e-commerce platforms that gather information or production companies that sell insects-based food in Europe.

### 2.2. Data Elaboration

Data about the products sold were recorded when consulting the available e-commerce platforms. Each producer was coded and associated with the country of production and their products. Data on the distribution of companies within each country, as well as the number and types of products, were collected and analysed to gain insight into the spread of different types of products across Europe. Countries were grouped according to the UN “Classification and definition of regions” [25] into Northern, Southern, Western, and Eastern. Data regarding products, including prices, insect species, percentage of insect inclusion in the formulations, and nutritional values, were collected from the ingredient lists and nutritional facts. This information was then used to establish a comprehensive dataset. The FoodEx2 classification [26] was used to group the products and gather them into commercial macro categories. Data about the nutritional composition such as calories (Kcal), carbohydrates, proteins, total fats, saturated fatty acids (SFAs), and salt content were recorded. To determine whether the available products could meet the daily nutritional needs of 97–98% of healthy individuals (National Institute of Health NIH), the Recommended Daily Allowance (RDA) was calculated based on 100g of each product. All product prices were converted to the price per kilogram (or Liter for beverages) in Euros, including those in different currencies (average exchange rate for 2023: 0.8700 EUR/GBP, 0.9720 EUR/CHF, 7.4536 EUR/DKK, 11.430 EUR/NOK, 0.04166 CZK/EUR, and 0.5111 BGN/EUR [27]).

## 3. Results and Discussion

### 3.1. Distribution of the Companies Across Europe

From data collection and through inclusion and exclusion criteria, 523 products proposed by fifty-three companies were found online. The production companies were located mostly in European Western (47.17%) countries, followed by Northern (26.42%), Eastern (15.09%), and Southern (11.32%) countries (Table 1). It is evident how the South of the continent still has an important under-representation of the insect food commerce. Consumers’ willingness to eat insects in Mediterranean Europe is very low as reported by Mancini et al. [28]. It could be related to the strong culinary culture and deep-rooted Mediterranean diet idea, as also highlighted, e.g., by the request of Italy to recognise its traditional cuisine as a UNESCO Intangible Cultural Heritage [29]. Following the data about the producer’s distribution, a wider market exists in Western (56.02% of products) and, immediately afterward, in Northern (18.93%), Eastern (15.11%), and Southern European countries (9.94%) (Table 1). Looking at the percentages reported above, it is possible to state that the distribution of food items in Europe faithfully reflects the companies’ distribution in the continent with a bigger market density in North–West countries. The results are in line with those of [23] which underlined a bigger presence of companies in Northern Europe, mostly in the United Kingdom. Indeed, as reported in Table 1, it is possible to confirm that the United Kingdom (8 companies) is the European country with the highest concentration of edible insect producers, followed by France (6), the Czech Republic (6), and Germany and Switzerland (both 5). With 60.61% of the total products, these five countries are the most active in the European insect food trade. Consumers can buy from French companies up to 115 different products (21.99% of the total). Czech Republic companies are the second one, with a wide offer of 69 products (13.19%), and the third are German companies with 67 products (12.81%). These companies compose a market that is still a niche in Western society, especially in Europe [30], but that represents a promising growing industry. It is forecasted to develop significantly in the next years (together with the North American one), with a good spread of iFBOs (Insect Food Business Operators) which, nowadays, are largely composed of micro companies (81%), followed by small (16%) and medium (3%) size companies [31]. In the past decade, in the edible insect market, several initiatives have transitioned, over time, from startups to well-established operators [17]. This process is creating many new jobs—not only direct ones (in the insect’s breeding sector or in the processing of insects for food), but also indirect ones (involved, for example, in the logistics or administrative side). In 2020, about 60% of the 33 European companies who have responded to a survey conducted by IPIFF—out of a total of 71 companies identified—had their respective national territory as a primary market target; in second place, products were sold in the European market, represented by Member States and EFTA (European free trade association) countries (Iceland, Liechtenstein, Norway, and Switzerland). The last market target was the international market, which includes non-EU and non-EFTA countries [32].

### 3.2. Insect Species and Products

**Figure 1:**
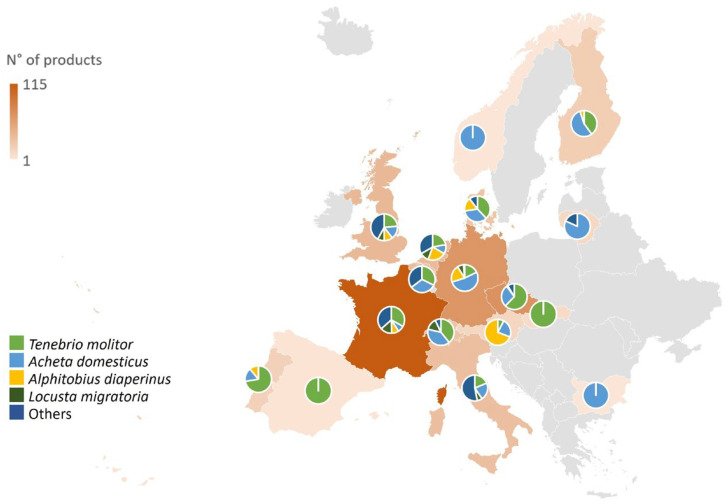
Distribution of products across Europe and prevalence of insect species per country.

In general, *Tenebrio molitor*-based products represent most of the products found in the European e-commerce marketplace (182 products), but there are differences between the different regions (Figure 1). Indeed, in Northern and Western regions, *Acheta domesticus*-based products are the most widespread type of products, while in Eastern and Southern regions, many of the available products are *Tenebrio molitor*-based (Figure 1). In some countries, i.e., Belgium, companies offer products just with two types of insects (mealworm and house cricket), or just one as in Spain and Slovakia (mealworm) and Norway and Bulgaria (house cricket). On the other hand, France and the United Kingdom have the widest choice of insect species (Figure 1). The differences in the products offered can be related to some consumers’ preconceptions about specific insect species. In fact, as reported by Fischer and Steenbekkers [33], people in Western countries mostly prefer crickets, mealworms, and grasshopper (*Locusta migratoria*) products or even the preparation of these products. The market can also be influenced by the ease of large-scale production of some species and their developmental stage [34,35]. Germany and Austria are the countries in which the offer of lesser mealworm-based products (54) is the widest (Figure 1).

This limited spread could be related to the fact that lesser mealworm can be seen as a pest which is a reservoir or vector of bacteria or mycotoxins [36,37]. Even though grasshopper is the most consumed whole insect species as reported by the IPIFF 2024 report, *Locusta migratoria*-based products are less prevalent than all the other insect products in all the European countries (34 products). In France, three of the seven existing companies produce about half of the products on the market with grasshopper. *Locusta migratoria* is regarded as a significant and detrimental pest for cultures. As reported by Van Peer et al. [38] numerous unsuccessful attempts have been made to develop an artificial diet for *Locusta migratoria.* Both could be compelling reasons for the limited availability of grasshopper products on the market. The ‘other’ category contains products based on species that have not yet seen an implementation of the Regulation (EU) 2015/2283. The availability of these products is contingent upon transitional measures that pertain to operators who were already engaged in commercial activities within the European Union before 1 January 2018 and who had submitted a formal request for authorisation. Towards the insect-based products involving the four species reported above, in the European e-commerce marketplace, it is possible to find 113 products prepared with 20 different species mainly represented by silkworms, superworms, bamboo worms, caterpillars, termites, ants, giant wasps, and giant water bugs. In this category, cricket-based products of species other than *Acheta domesticus* were also included, such as *Gryllus assimilis*, *Grylloides sigillatus*, and *Gryllotalpa gryllotalpa*. Products without the scientific name of the species, but with the common names, on the packaging were also added to this list. Interestingly, for 90 products without the scientific names of the insect species, 70 were cricket-based. Products were available in different formulations (Table 2): whole insect, insect powder, protein products, meat products, meat substitutes, bakery products and premixes, cereal, biscuits and bars, pasta products (dried), chocolate products, snacks of various types, fermented and non-fermented alcoholic beverages, and others. The most abundant products found in the e-commerce marketplace belong to the ‘whole insect’ category, followed by the ‘sweet and candies’ and ‘insect powder’ categories (Table 2). Although insects in whole form are less easily accepted by consumers [39], whole insects represent the most readily available macro category comprising 50.67% of the total. The lack of visibility of the insect could help to minimise the disgust factor [40], and to familiarise European consumers with edible insect products, the remaining 49.33% of the available products are represented by insects in powder form, to enrich foods such as pasta, crackers, or bars. Insect-based ‘protein products’ represent 2.10% of the market and are composed primarily of ingredients derived from the lesser mealworm (*Alphitobius diaperinus*) and the house cricket (*Acheta domesticus*). ‘Meat products’ are the scarcer, with only two products found that are composed of 50% beef meat and 50% insects. As evidenced by the studies conducted by Kim et al. [41,42], the incorporation of insect ingredients reduced moisture loss in meat emulsions. Likely, consumers are not yet prepared to accept this type of product, and companies are therefore testing the commercial viability of these two innovative products, which contain *Tenebrio molitor* larvae and crickets. ‘Bakery products and premixes’ represent 5.54% of the total products, and the most abundant products contain *Tenebrio molitor* (Table 2). The incorporation of insects into bakery products has been demonstrated to enhance the nutritional value of the products, particularly in terms of protein content [43]. As with bakery products, *Tenebrio molitor* and *Acheta domesticus* were the most abundant, followed by *Alphitobius diaperinus* and other species. ‘Pasta products’ represent 3.06% of the total, and it is possible to find the same amount of *Tenebrio molitor* and *Alphitobius diaperinus* pasta. As demonstrated by Duda et al. [44], the incorporation of insect powder in pasta did not show significant sensory differences. Of the 523 products, 32 are chocolate-based, with mealworm-based products representing much of this category. ‘Snacks of various types’, namely chips and tortillas, represent 4.21%, and they are prepared primarily with house crickets, other crickets, and mealworms ingredients. ‘Alcoholic beverages fermented and non-fermented’ (1.15%) are represented by aromatised vodka and beer. These are prepared mainly with mealworms. The ‘others’ category contains savoury sauces such as hummus and tapenade and peanut butter which represent 0.96% of the total products range.

### 3.3. Level of Insect Inclusion, Prices and Packaging

Table 3 shows the percentages of inclusion of insects in the various products, the prices, and the quantities. Insect powders consisted of 100% insects, while in the case of whole insect products, if flavoured with salt and spices, the percentages dropped to 70–75%. Products differ also in weight and, consequently, in price. Whole insect products can be purchased in packs containing from 5 to 50 g of product, while larger packages of whole insects (from 200 g to 1 kg) are designed for sale to the catering sector. Whole and flavoured insects are mainly intended to be eaten as a snack and are enriched with spices that allow the Asian culinary tradition, which has integrated entomophagy for centuries, to be mixed with the flavours of other traditions and spice up the curiosity of European consumers. The powder formulations of buffalo worms, mealworms, house crickets, and grasshoppers were available in packs of 100 g, 200 g, 500 g, or 1 kg. In this way, they can be stored like common wheat flour and be used for domestic preparation. Our results showed that the average price in EUR/Kg for grasshopper’s whole insects and powder products were the highest (Table 3). When the insect—in pieces or powder—was employed in the formulation of bars, pasta, or crackers, for example, the percentage of inclusion was quite low, between 14% (as in pasta with lesser mealworm) and almost negligible percentages such as 5.6% for chocolate grasshoppers. In general, if compared to other conventional products, prices for insect-based products are often higher. For example, if the price of house cricket powder is compared with that of common durum wheat semolina, the first has a cost of 149.92 EUR/Kg and the second of 0.52 EUR/Kg [45]. Due to its different composition, insect powder cannot completely replace wheat flour but can be used to increase the protein content of carbohydrate-based food products as demonstrated by Duda et al. [44]. Indeed, to improve the nutritional value of pasta, they made three formulations of spaghetti with durum wheat semolina and cricket powder in ratios of 95:5, 90:10, and 85:15. Pasta containing insect powder resulted in enrichment of proteins, lipids, and minerals and depletion of carbohydrates proportionally to the increase in cricket powder used. They also registered changes regarding the colour of spaghetti (darker with the inclusion of cricket powder), the cooking time, and the firmness after cooking (which increases as the percentage of cricket powder increases). A greater firmness gives the product compactness appreciated by consumers, unlike the dark colour which would seem to discourage the consumption of pasta. Even García-Segovia et al. [46] showed that regardless of the species and amount of *Tenebrio molitor* and *Alphitobius diaperinus* powder used to replace 5% and 10% of soft wheat flour in bread, the rheological properties of the dough as an index of extensibility and swelling are not modified. As reported, the perception of consumers is influenced positively by high prices which were associated with high-quality products [47]. Hard candies, especially lollipops, always contained a whole single insect inside; as for alcohol, these products focused a lot on the visual effect, here given by the bright dyes and the presence of whole arthropods. In alcoholic products, the percentage of alcohol varies between 37.5 and 40%, while the bottles available were designed for a single size or are larger, from 100 to 750 mL. Prices should adapt to those of conventional products to be more competitive in the market [48]. Looking at product packaging, there is a noticeable trend where the use of vibrant, eye-catching colours stands out, and it is interesting to note that these packages often do not display images of the insects used in the products’ formulation. Instead, they typically include visual references to other ingredients opting to emphasise its nutritional contribution through descriptive language and the ingredients list. Many companies place a strong emphasis on clearly communicating the high protein content that insects offer, underscoring the significance of proteins as the most prevalent nutrients in the nutritional profile of edible insects [49]. Furthermore, some producers display food certifications on the packaging as proof of the high quality and safety of the products [50].

### 3.4. Nutritional Value

The minimum, maximum, and average values for the nutritional values (in terms of Kcal, macronutrients, and micronutrients) referring to 100 g of product are reported in Table 3.

#### 3.4.1. Energy Content

Edible insect-based products provide a satisfactory share of energy and protein, containing a good amount of mono and polyunsaturated fatty acids, micronutrients (such as vitamins riboflavin, pantothenic acid, biotin, cobalamin, and ascorbic acid), and mineral salts (e.g., microelements such as iron and selenium and macroelements such as phosphorus and magnesium) [14]. The analysis of 78 insect species conducted by Ramos-Elorduy [51] revealed an energy content ranging from 293 to 762 kcal per 100 g of dry matter, mostly attributable to protein content. As shown in Kouřimská and Adámková [52], lipid content also plays an important role in determining the total energy content of insects. It is important to underline that the nutritional profile, in this specific case being energy, is subject to important variations that can occur both between different species and between individuals of the same species (which are in a different metamorphic stage or have been fed with a different feed) [53,54]. In Table 3, the products with the highest Kcal content were in the ’chocolate and similar’ category for all species except grasshoppers, which had the highest Kcal value for ’insect powder’. While the lowest Kcal values were ‘savory sauces’ for mealworms and other species, ‘hard candies’ for crickets, and ‘meat imitates’ for lesser mealworms. The %RDA Kcal values for 100 g of ’insect powder’ and ‘whole insect’ ranged between 29.75% for the grasshopper powder and 21.30% for the whole house cricket (Table 4). Therefore, 100 g of products, mostly composed of insects, could contribute from one-third to one-fifth of the recommended daily allowance of energy. Interestingly, 100 g of *Tenebrio molitor* ‘dried pasta’ provides an energy content of 387.6 Kcal (Table 3), which is higher than the 341 Kcal/100 g of conventional pasta [55]. Therefore, comparing the %RDA Kcal values of the *Tenebrio molitor* pasta, the insect-based food resulted more energetic (19.36% and 17.05%, respectively, for mealworm and conventional pasta). This could be related to the protein content of *Tenebrio molitor* pasta which is significantly higher than the protein content of pasta (19.24 g and 13.5 g, respectively).

#### 3.4.2. Protein Content

A noteworthy aspect of the nutritional profile of these products is the commendable protein content, which is highest in whole insects and decreases in processed products (e.g., biscuits, pasta, and crackers) in proportion to the percentage of insect inclusion (Table 3). Feng et al. [56] reported that the protein content of insects constitutes approximately 50% of the total weight of insects. Analysing 236 insect species, Rumpold and Schlüter [49] reported that protein content in different insect orders varies, ranging from an average of 35.34% for the order Isoptera to an average of 61.32% on a dry matter basis for the order Orthoptera. Similarly, Xiaoming et al. [57], analysing 100 species from all the insect orders, established a wider range (13–77% of proteins on a dry matter basis). The nutritional composition of the products (Table 3) showed that the highest average protein content belonged to the lesser mealworm ‘isolated proteins and other protein products’, followed by the other species ‘isolated proteins and other protein products’ and house cricket powder. On the other hand, the lowest values were registered in all species for ‘hard candies’. Introducing insect-based products into the diet, and in particular whole and powdered insects, can amply satisfy the recommended daily protein intake. Indeed, Table 4 reveals that 100 g of *Acheta domesticus* powder provides 67.53 g of proteins, corresponding to 90.03% of the average nutrient intake levels given in the RDAs. The highest ‘whole insect’ protein content in 100 g was in lesser mealworm with 59.25 g of protein equal to 79.00% of the RDA.

#### 3.4.3. Carbohydrates and Sugars Content

The exoskeleton of insects is mainly composed of carbohydrates, even if their quantity in the whole composition of the insect body, compared to fat and protein, is low [58]. In the reviewed ‘meat burgers’ and ‘preserved and partially preserved sausages’ categories, the carbohydrate portion is almost negligible (Table 3). A low level of carbohydrates could also be found in both ‘whole insects’ and ‘insects powder’ (Table 3). As expected, high carbohydrate content products were ‘dried pasta’, ‘biscuits’, and ‘dry premixes for baked products’, these values could be mainly ascribed to the other ingredients present in the original recipes. Similar results were reported for sugar content for ‘chocolate and similar’ products and in lesser mealworm ‘sweet bars and other sweet masses’ products. Hard candies had the highest carbohydrate content and %RDA on a 100 g portion because they are made of sucrose, a low-molecular-weight carbohydrate. Sugar content is very low in ‘insects powder’ with values near to 0. The %RDA follow the same trends as reported Table 4.

#### 3.4.4. Lipid Content

The content of saturated fatty acids is specified for most products, while there are not always indications referring to mono- and polyunsaturated fatty acids (MUFAs and PUFAs). Despite this, there is evidence from the literature that the predominant component of the lipid profile of the species employed in insect-based food is made up of unsaturated fatty acids (around 65%, in which MUFAs represent the 40% and PUFAs 25%) [59]. In general, the saturated fatty acids most present in the composition of insect lipids are palmitic acid (C16:0) and stearic acid (C18:0); oleic acid (C18:1) is the most represented MUFA, while linoleic (C18:2n6) and linolenic (C18:3n3) fatty acids represent the majority of PUFAs [53]. Tzompa-Sosa et al. [60] reported that eicosapentaenoic acid (EPA, C20:5n3) and docosahexaenoic acid (DHA, C22:6n3) are present only in some species and not in elevated quantities. The fattiest product was the lesser mealworm ’peanut butter’, followed by the lesser mealworm and mealworm ’chocolate and similar’ products, which also had higher saturated fatty acids (SFA) content due to the natural fat content of chocolate (Table 3). Therefore, the %RDA is affected by the ingredient list, e.g., the house cricket ‘biscuits’ that contains from 6 to 10% insect have a total fat of 35.60 g / 100 g portion, which corresponds to 45.64% of the RDA of total fats (Table 4). The fatty acids profile of each insect species affect the nutritional values of the products. Indeed, as it is widely reported that mealworm and grasshopper have a high content of total fat (on dry bases), *Tenebrio molitor* is particularly rich in MUFA and oleic acid, while *Locusta migratoria* has a high content of SFA [59,60,61,62]. These characteristics reflect the high content of both fat per 100 g of mealworm products (30.23 g ‘insect powder’ and 28.43 g ‘whole insect’) and grasshopper products (38.1 g for ‘insect powder’ and 24.42 g for ‘whole insect’). Then, due to the fatty acids profile of the two species, the SFA %RDA for mealworm products are 75.08% and 81.56% (respectively, for ‘insect powder’ and ‘whole insect’) and 163.75% for grasshopper powder and 100.69% for 100 g of whole grasshopper (Table 4).

#### 3.4.5. Salt and Fibre Content

The World Health Organization recommends that adults should consume less than 2 g of sodium (5 g of salt) per day [63]. Products with the lowest average amount of salt were lesser mealworm ‘muesli and similar mixed breakfast cereals’ and mealworm ‘cereal bars’ and ‘dried pasta’ (Table 3). While products with the highest average salt content in 100 g of products were other species in the ‘preserved and partially preserved sausages’ category and ‘whole’ house crickets. Insect fibres are mainly represented by chitin, a long-chain polymer of N-acetyl-glucosamine that can regulate human and animal gut microbiota, improving health [64]. Products with the lowest fibre content were mealworm ‘biscuits’, and those with the highest were other species ‘sweet bars and other sweet masses’ and ‘crackers and breadsticks’ and house crickets ‘chips/crisps’ (Table 3). Nevertheless, insect-based products have an excellent fibre content which help to achieve the recommended 25 g of fibre per day [65].

## 4. Conclusions and Future Perspectives

The available edible insect-based products in the European e-commerce market are mainly snacks, chocolate, and similar products. These categories of products are well-known by European consumers and present the insect in an invisible way (powder); moreover, they can be linked to pleasant taste experiences. They are undoubtedly the easiest form to accept and consume edible insects but also the most difficult to include in daily diets. Therefore, these categories could be considered as occasionally experimental consumption products. To overcome negative attitudes towards insect-based food, it is important to raise the consumers’ awareness of the health benefits derived from insect consumption. An inclusion of insects in common food could increase the protein as well as the energy and fat contents affecting the nutritional value of the final product. Of note, this depends on the product typology, insect species, processing, and the quantity of insects included in the food. It would be beneficial to focus future efforts from scientists and producers on divulging accurate information about the potential health benefits of incorporating edible insects into diets. Furthermore, to reach a wider segment of the population, a tailormade communication strategy should be developed suggesting the consumption of insects through social media, e.g., proposing catching recipes and breaking down the misconception against entomophagy. The producer’s mission should include a wider variety of ingredients in product formulations, both with plant- and animal-based products, not only focusing on already employed species but increasing the diversification of the productions. In particular, for this sector, they should explore and test various recipes in order to enhance the variety of insect-based food products. Policymakers should focus on developing policies that support and promote a sustainable transition to a green economy. In relation to the insect sector, specific regulations are attended in order to increase the exploitation of the true potential of edible insects. Meanwhile, consumers should go beyond their established habits and try to change their lifestyles by choosing goods that come from sustainable production. All those actions, among others, could contribute to the advancement of the insect sector, encouraging the development of innovative new products and ingredients that could help to reinforce the insect producers’ position as a promising avenue for the future of the food system.

## Figures and Tables

**Table 1:** Insect food products sold online in the Europe continent.

Country	Companies %	N° *Tenebrio* *molitor*	N° *Acheta* *domesticus*	N° *Alphitobius* *diaperinus*	N° *Locusta* *migratoria*	N° Other Species	N° Products	Products %
Eastern								
Bulgaria	1.89%	-	1	-	-	-	1	0.19%
	a	-	1	-	-	-	1	
Czech Republic	11.32%	43	19	1	-	6	69	13.19%
	a	10	-	-	-	5	15	
	b	13	3	-	-	1	17	
	c	7	6	-	-	-	13	
	d	13	-	-	-	-	13	
	e	-	7	1	-	-	8	
	f	-	3	-	-	-	3	
Slovakia	1.89%	9	-	-	-	-	9	1.72%
	a	9	-	-	-	-	9	
Northern								
Denmark	5.66%	11	10	5	-	3	29	5.54%
	a	11	-	-	-	-	11	
	b	-	-	5	-	3	8	
	c	-	10	-	-	-	10	
Finland	1.89%	8	11	1	-	-	20	3.82%
	a	8	11	1	-	-	20	
Lithuania	1.89%	-	9	-	-	2	11	2.10%
	a	-	9	-	-	2	11	
Norway	1.89%	-	1	-	-	-	1	0.19%
	a	-	1	-	-	-	1	
UK	15.09%	9	6	4	3	16	38	7.27%
	a	-	3	-	-	-	3	
	b	-	1	-	-	-	1	
	c	6	-	1	1	1	9	
	d	1	-	1	1	7	10	
	e	2	-	2	1	1	6	
	f	-	1	-	-	-	1	
	g	-	1	-	-	4	5	
	h	-	-	-	-	3	3	
Southern								
Italy	7.55%	6	7	-	2	17	32	6.12%
	a	1	3	-	-	-	4	
	b	5	-	-	-	-	5	
	c	-	1	-	2	17	20	
	d	-	3	-	-	-	3	
Portugal	1.89%	13	3	2	-	-	18	3.44%
	a	13	3	2	-	-	18	
Spain	1.89%	2	-	-	-	-	2	0.38%
	a	2	-	-	-	-	2	
Western								
Austria	1.89%	1	3	9	-	-	13	2.49%
	a	1	3	9	-	-	13	
Belgium	7.55%	11	11	-	-	12	34	6.50%
	a	4	-	-	-	-	4	
	b	1	-	-	-	-	1	
	c	-	11	-	-	1	12	
	d	6	-	-	-	11	17	
Germany	9.43%	12	35	14	5	1	67	12.81%
	a	-	4	-	-	-	4	
	b	3	11	-	1	1	16	
	c	-	-	7	-	-	7	
	d	8	13	7	3	-	31	
	e	1	7	-	1	-	9	
France	11.32%	38	9	10	16	42	115	21.99%
	a	10	2	-	-	2	14	
	b	9	2	-	1	2	14	
	c	-	-	-	-	5	5	
	d	7	4	10	8	-	29	
	e	7	-	-	7	24	38	
	f	5	1	-	-	9	15	
The Netherlands	7.55%	8	4	8	4	12	36	6.88%
	a	-	3	1	-	-	4	
	b	4	1	2	2	3	12	
	c	-	-	-	-	4	4	
	d	4	-	5	2	5	16	
Switzerland	9.43%	11	11	-	4	2	28	5.35%
	a	-	3	-	-	-	3	
	b	9	3	-	1	2	15	
	c	1	4	-	3	-	8	
	d	1	-	-	-	-	1	
	e	-	1	-	-	-	1	
Total								
17	53	182	140	54	34	113	523	

**Table 2:** Main product commercial categories.

Commercial Categories	FoodEx2 Codes	Food Category	N° Products	% of Products	N° *Tenebrio molitor*	N° *Acheta* *domesticus*	N° *Alphitobius* *diaperinus*	N° *Locusta* *migratoria*	N° Other Species
Whole insect	A06HL	Snacks other than chips and similar	265	50.67%	93	73	12	28	59
Sweet and candies	A034X	Hard candies	65	12.43%	20	11	13	3	18
	A0EQR	Sweet bars and other formed sweet masses							
Insect powder	A06HL	Snacks other than chips and similar	36	6.88%	11	8	6	2	9
Chocolate products	A0EQD	Chocolate and similar	32	6.12%	16	7	1	1	5
Bakery products and premixes	A005Y	Crackers and breadsticks	29	5.54%	13	9	4	-	3
	A0CSK	Pre-mixes (dry) for baked products							
Cereal, biscuits and bars	A009V	Biscuits	29	5.54%	11	11	6	-	1
	A00EY	Cereal bars							
	A00EJ	Muesli and similar mixed breakfast cereals							
	A00EN	Porridge							
Snacks of various types	A0EQX	Chips/crisps	22	4.21%	6	9	-	-	7
	A00FD	Tortilla chips							
Pasta products (dried)	A007L	Dried pasta	16	3.06%	5	4	5	-	2
Protein products	A0EVD	Isolated proteins and other protein products	11	2.10%	-	2	5	-	4
Meat substitutes	A03TE	Meat imitates	7	1.34%	1	5	1	-	-
Fermented and non-fermented alcoholic beverages	A03MA	Beer	6	1.15%	3	1	-	-	1
	A03PD	Unsweetened spirits							
Others	A01BN	Peanut butter	5	0.96%	2	-	1	-	2
	A043V	Savoury sauces							
Meat products	A03XF	Meat burger	2	0.38%	1	-	-	-	1
	A0EYP	Preserved or partly preserved sausages							
		Total	523		182	140	54	34	113

**Table 3:** Nutritional values of the insect-based food products.

FoodEx2 Code	Food Category	N° Products	Average Price	Insect %	Kcal	Proteins	Carbohydrates	Sugars	Total Fats	SFA	Salt	Fiber
Mealworm (*Tenebrio molitor*)												
A03MA	Beer and beer-like beverage	1	NA	NA	NA	NA	NA	NA	NA	NA	NA	NA
A009V	Biscuits	8	60.70	4.8–6.0	391.0–472.0 (434.2)	9.1–9.9 (9.6)	57.0–65.0 (59.8)	22.0–22.0 (22.0)	14.0–21.0 (17.3)	0.7–1.7 (1.2)	0.40–1.50 (0.90)	1.1–3.0 (2.1)
A00EY	Cereal bars	2	72.00	10.0–10.0	462.4–468.0 (465.2)	14.7–15.8 (15.3)	43.9–50.3 (47.1)	27.0–27.0 (27.0)	21.1–23.0 (22.1)	2.9–4.4 (3.7)	0.07–0.08 (0.07)	5.9–7.4 (6.7)
A0EQX	Chips/crisps	5	101.20	10.0–18.0	417.0–472.0 (451.0)	8.3–15.0 (11.0)	55.0–61.0 (58.8)	0.6–3.3 (1.3)	14.0–21.2 (18.3)	2.1–2.4 (2.2)	2.10–2.80 (2.60)	2.2–5.2 (3.2)
A0EQD	Chocolate and similar	16	120.89	0.2–50.0	503.0–575.0 (542.2)	5.7–32.0 (10.9)	29.0–55.6 (40.5)	4.0–54.7 (31.1)	31.0–41.9 (37.2)	4.7–25 (17.1)	0.03–4.00 (1.20)	6.5–14.0 (11.0)
A005Y	Crackers and breadsticks	9	72.49	10.0–10.0	346.0–618.0 (463.4)	12.0–23.9 (18.2)	2.0–78.0 (47.1)	0.7–5.1 (2.2)	3.0–42.0 (20.2)	1.1–15 (7.9)	1.10–2.90 (1.80)	2.2–11.0 (5.1)
A007L	Dried pasta	5	32.20	10.0–10.0	386.2–391.0 (387.1)	18.8–21.0 (19.2)	56.1–62.0 (57.3)	3.1–5.0 (3.5)	6.3–7.9 (7.6)	1.9–1.9 (1.9)	0.09-0.09 (0.09)	7.7–9 (8.0)
A034X	Hard candies	14	191.88	NA	234.0-234.0 (234.0)	1.0-1.0 (1.0)	95.0-95.0 (95.0)	0.0-0.0 (0.0)	1.0-1.0 (1.0)	1.0-1.0 (1.0)	0.10-0.10 (0.10)	1.0-1.0 (1.0)
A03XF	Meat burgers	1	49.76	50.0–50.0	263.0-263.0 (263.0)	19.7-19.7 (19.7)	1.3-1.3 (1.3)	1.3-1.3 (1.3)	19.4-19.4 (19.4)	6.6-6.6 (6.6)	0.60-0.60 (0.6)	NA
A03TE	Meat imitates	1	36.00	30.0–30.0	275.0-275.0 (275.0)	16.6-16.6 (16.6)	19.6-19.6 (19.6)	3.8-3.8 (3.8)	13.5-13.5 (13.5)	2.4-2.4 (2.4)	1.30-1.30 (1.30)	4.6-4.6 (4.6)
A00EJ	Muesli and similar mixed breakfast cereals	1	23.33	NA	431.0-431.0 (431.0)	12.0-12.0 (12.0)	51.4-51.4 (51.4)	17.9-17.9 (17.9)	17.7-17.7 (17.7)	7.2-7.2 (7.2)	0.20-0.20 (0.20)	8.4-8.4 (8.4)
A0CSK	Pre-mixes (dry) for baked products	4	59.18	NA	475.0–618.6 (546.7)	20.1–23.9 (22.0)	29.0–34.2 (31.6)	0.7–1.0 (0.9)	26.2–42.0 (34.1)	2.9–7.9 (4.5)	0.50–1.10 (0.90)	7.9–11.0 (9.5)
A043V	Savory sauces	2	34.59	4.0–5.0	69.8–200.9 (135.4)	3.4–4.1 (3.8)	5.7–9.0 (7.4)	3.7–6.6 (5.2)	3.1–15.4 (9.3)	0.5–2.0 (1.3)	0.20–0.50 (0.40)	2.1–2.2 (2.2)
A06HL	Snacks other than chips and similar–insect powder	11	143.77	100.0	473.0–550.0 (507.7)	45.1–59.6 (54.2)	0.9–6.7 (4.1)	0.0–2.0 (0.5)	25.0–37.3 (30.2)	6.34–9.0 (7.7)	0.00–9.90 (1.80)	0.5–7.1 (4.6)
A06HL	Snacks other than chips and similar–whole insect	93	260.53	12.0–100.0	417.0–581.0 (495.2)	14.2–60.3 (48.9)	0.0–41.0 (8.9)	0.0–28.4 (2.1)	14.5–43.0 (28.4)	1.7–14.5 (6.9)	0.00–10.50 (2.80)	0.5–15.0 (6.2)
A0EQR	Sweet bars and other formed sweet masses	6	53.94	6.3–10.0	428.0–541.0 (452.5)	15.0–27.0 (20.0)	25.0–50.0 (36.2)	18.0–30.0 (24.3)	17.0–35.0 (23.5)	1.8–6.2 (3.4)	0.10–1.19 (0.30)	4.4–12.0 (7.9)
A00FD	Tortilla chips	1	77.13	38.0–38.0	244.0-244.0 (244.0)	13.8-13.8 (13.8)	36.2-36.2 (36.2)	2.2-2.2 (2.2)	6.6-6.6 (6.6)	1.6-1.6 (1.6)	2.20-2.20 (2.20)	3.4-3.4 (3.4)
A03PD	Unsweetened spirits	2	101.80	NA	NA	NA	NA	NA	NA	NA	NA	NA
House cricket (*Acheta domesticus*)												
A009V	Biscuits	5	80.04	6.0–10.0	431.0–598.0 (537.0)	11.3–18.0 (15.4)	28.0–54.3 (36.2)	1.0–30.8 (12.3)	24.1–39 (35.6)	6.0–21.9 (10.8)	0.30–1.00 (0.80)	3.2–3.2 (3.2)
A00EY	Cereal bars	3	61.68	5.0–5.0	388.0–434.0 (407.3)	14.0–15.4 (14.5)	31.0–41.4 (35.5)	6.9–13.1 (9.4)	17.0–25.4 (20.5)	2.6–8.6 (4.7)	0.04–0.44 (0.23)	8.0–28.0 (21.0)
A0EQX	Chips/crisps	9	51.9	7.0–7.0	338.0–393.9 (375.5)	23.0–25.1 (24.5)	41.2–51.0 (44.9)	3.1–9.8 (4.6)	10.0–14.9 (13.1)	1.6–2.2 (1.9)	2.60–2.90 (2.70)	8.3–23.0 (13.6)
A0EQD	Chocolate and similar	7	159.33	40.0–56.0	415.0–587.3 (522.2)	6.3–37.0 (23.5)	16.0–39.0 (29.0)	14.0–38.4 (28.6)	26.0–41.1 (33.2)	17.0–24.0 (19.5)	0.10–4.00 (0.86)	8.1–8.1 (8.1)
A005Y	Crackers and breadsticks	8	49.93	12.0–17.0	399.5–507.0 (458.2)	21.0–25.4 (22.1)	20.0–63.1 (41.4)	1.0–5.6 (3.4)	8.1–36.0 (21.5)	1.4–6.3 (3.2)	1.60–3.00 (2.27)	3.3–12.0 (7.6)
A007L	Dried pasta	4	23.84	15.0–17.0	321.0–360.0 (345.8)	19.6–41.0 (35.2)	32.0–58.2 (40.8)	0.4–2.3 (1.08)	4.5–6.3 (5.5)	1.5–1.9 (1.7)	0.00–1.00 (0.7)	3.5–5.1 (4.3)
A034X	Hard candies	2	149.5	NA	234.0-234.0 (234.0)	1.0-1.0 (1.0)	95.0-95.0 (95.0)	0.0-0.0 (0.0)	1.0-1.0 (1.0)	1.0-1.0 (1.0)	0.10-0.10 (0.10)	1.0-1.0 (1.0)
A0EVD	Isolated proteins and other protein products	2	63.76	5.1–10.0	361.0–375.0 (368.0)	32.0–74.0 (53.0)	3.5–33.0 (18.3)	0.1–2.7 (1.4)	5.2–16.0 (10.6)	1.7–12.0 (6.85)	0.20–2.80 (1.50)	7.2–7.7 (7.5)
A03TE	Meat imitates	5	38.29	20.0-20.0	219.0–407.0 (293.8)	15.2–77.0 (40.3)	0.0–9.9 (4.7)	0.0–0.69 (0.3)	10.0–13.5 (11.4)	2.6–10.0 (6.6)	1.32–1.80 (1.54)	NA
A00EJ	Muesli and similar mixed breakfast	3	19.97	5.0-5.0	432.8–451.9 (443.0)	12.4–14.6 (13.7)	47.9–52.3 (49.5)	12.4–16.3 (13.9)	18.9–21.1 (20.2)	2.3–5.5 (3.5)	0.06–0.60 (0.25)	8.4–10.6 (9.1)
A0CSK	Pre-mixes (dry) for baked products	1	22.86	15.0-15.0	336.0–336.0 (336.0)	22.0-22.0 (22.0)	55.0-55.0 (55.0)	0.8-0.8 (0.8)	4.5-4.5 (4.5)	1.5-1.5 (1.5)	1.92-1.92 (1.92)	4.9-4.9 (4.9)
A06HL	Snacks other than chips and similar—insect powder	8	108.01	100.0-100.0	400.0–505.0 (448.0)	63.0–75.9 (67.52)	0.5–18.0 (6.9)	0.0–0.6 (0.14)	6.8–26.4 (17.3)	2.3–10.3 (6.4)	0.50 -0.80 (0.72)	4.0–9.5 (6.2)
A06HL	Snacks other than chips and similar—whole insect	73	316.44	40.9–100.0	291.0–525.0 (444.2)	22.2–72.4 (53.5)	0.0–51.2 (9.0)	0.0–51.1 (3.75)	7.2–33.0 (22.3)	2.7–12.4 (7.9)	0.30–9.61 (3.06)	0.9–18.0 (5.1)
A0EQR	Sweet bars and other formed sweet masses	9	53.23	10.0–20.0	356.0–535.0 (424.3)	12.7–33.3 (22.0)	10.0–42.9 (26.8)	7.2–42.6 (20.64)	14.0–36.5 (21.9)	5.8–12.2 (9.4)	0.07–0.45 (0.24)	2.7–30.0 (14.1)
A03PD	Unsweetened spirits	1	111.10	NA	NA	NA	NA	NA	NA	NA	NA	NA
Lesser mealworm (*Alphitobius diaperinus*)												
A0EQD	Chocolate and similar	1	69.08	NA	580.0-580.0 (580.0)	8.8-8.8 (8.8)	35.0-35.0 (35.0)	29.0-29.0 (29.0)	43.0-43.0 (43.0)	26.0-26.0 (26.0)	0.25-0.25 (0.25)	NA
A007L	Dried pasta	5	18.58	3.9–14.0	371.0–374.0 (373.2)	15–18.7 (17.8)	64.0–72.0 (66.2)	1.8–3.2 (2.74)	1.9–4.4 (3.74)	0.8–1.2 (1.02)	0.10–0.13 (0.12)	2.0–3.8 (3.4)
A034X	Hard candies	1	149.5	NA	234.0-234.0 (234.0)	1.0-1.0 (1.0)	95.0-95.0 (95.0)	0.0-0.0 (0.0)	1.0-1.0 (1.0)	1.0-1.0 (1.0)	0.10-0.10 (0.10)	NA
A0EVD	Isolated proteins and other protein products	5	60.79	20.0–35.0	416.0–437.6 (428.7)	68.5–71.5 (70.2)	3.43–11.1 (6.5)	0.6–8.3 (3.6)	10.0–17.8 (14.2)	4.0–6.92 (4.9)	0.30–1.05 (0.92)	3.0–7.1 (5.3)
A03TE	Meat imitates	1	15.00	24.9-24.9	195.0-195.0 (195.0)	15.6-15.6 (15.6)	5.2-5.2 (5.2)	0.3-0.3 (0.3)	9.7-9.7 (9.7)	6.4-6.4 (6.4)	1.70-1.70 (1.70)	NA
A00EJ	Muesli and similar mixed breakfast cereals	4	23.96	5–10	391.0–453.0 (413.0)	13.0–14.0 (13.7)	45.2–57.0 (52.8)	15.0–22.2 (17.1)	11.0–22.6 (15.15)	2.1–4.1 (2.75)	0.05–0.13 (0.09)	4.1–7.3 (5.2)
A01BN	Peanut butter	1	19.94	15.0–15.0	582.0-582.0 (582.0)	31.5-31.5 (31.5)	8.9-8.9 (8.9)	6.2-6.2 (6.2)	46.8-46.8 (46.8)	6.9-6.9 (6.9)	0.42-0.42 (0.42)	7.6-7.6 (7.6)
A00EN	Porridge (dry form—to be diluted)	2	32.38	20.0–20.0	395.8–411.8 (403.8)	20.4–21.4 (20.9)	41.8–45.2 (43.5)	15.3-15.3 (15.3)	12.86–15.38 (14.12)	4.8–5.7 (5.3)	0.25-0.25 (0.25)	10.3-10.3 (10.3)
A0CSK	Pre-mixes (dry) for baked products	4	25.46	5.5–12.0	342.0–369.0 (356.5)	11.0–23 (16.5)	44.0–69.0 (58.0)	4.1–40.0 (16.0)	2.6–7.9 (4.725)	0.5–1.3 (1.0)	0.04–4.40 (1.58)	NA
A06HL	Snacks other than chips and similar—insect powder	6	122.23	99.9–100.0	510.0–511.0 (510.5)	59.6–62.0 (60.8)	2.7–8.0 (5.4)	0.5–0.5 (0.5)	24.3–29 (27)	8.4–10.1 (9.6)	0.50–0.90 (0.71)	3.7–7.0 (5.6)
A06HL	Snacks other than chips and similar—whole insect	12	265.55	100.0-100.0	484.0–510.0 (501.0)	56.3–60.0 (59.3)	2.6–6.7 (3.8)	0.5–0.7 (0.62)	24.2–28.7 (27.06)	9.4–10.1 (9.6)	0.01–3.60 (1.03)	3.3–5.2 (4.0)
A0EQR	Sweet bars and other formed sweet masses	12	76.72	5.0–13.0	343.0–503.0 (408.2)	13.0–20.0 (16.7)	33.0–55.0 (42.6)	29.0–44.0 (37.7)	4.5–31 (16.76)	1.4–5.7 (3.0)	0.10–0.50 (0.23)	5.3–11.0 (8.8)
Grasshopper (*Locusta migratoria*)												
A0EQD	Chocolate and similar	1	790.00	59.0-59.0	371.0-371.0 (371.0)	43.0-43.0 (43.0)	21.0-21.0 (21.0)	17.0-17.0 (17.0)	10-10 (10)	4-4	NA	NA
A034X	Hard candies	3	NA	NA	NA	NA	NA	NA	NA	NA	NA	NA
A06HL	Snacks other than chips and similar—insect powder	2	499.00	100.0	595.0-595.0 (595.0)	48.2-48.2 (48.2)	NA	NA	NA	NA	NA	NA
A06HL	Snacks other than chips and similar—whole insect	28	614.00	56.5–100.0	322.0–599.0 (454.8)	24.9–70.0 (46.03)	0.1–24.0 (6.9)	0.0–2.4 (1.5)	11–38.1 (24.95)	0.0–9.8 (8.1)	0.20–40 (1.24)	6.5–11.5 (8.3)
Other species												
A009V	Biscuits	1	84.22	NA	NA	NA	NA	NA	NA	NA	NA	NA
A0EQX	Chips/crisps	7	58.65	8.0–10.0	399.0–476.3 (434.5)	20.0–24.0 (21.5)	40.7–50.6 (44.8)	1.8–3.9 (3.0)	10.1–19.1 (15.3)	1.4–1.7 (1.6)	0.40–3.70 (1.94)	8.8–14.6 (12.1)
A0EQD	Chocolate and similar	5	113.79	8.0-8.0	420.0–587.3 (503.1)	6.3–16.6 (13.2)	30.6–41.0 (38.1)	27.6–38.4 (35.1)	19.6–41.1 (30.58)	5.7–24 (15.5)	0.10–0.38 (0.25)	8.9–8.9 (8.9)
A005Y	Crackers and breadsticks	3	40.06	8.0–10.0	383.0–495.0 (420.3)	23.0–25.1 (24.4)	23.0–39.0 (33.7)	1.0–2.7 (2.1)	12.7–32 (19.13)	1.9–4.8 (2.9)	1.20–2.90 (2.33)	12.0–14.3 (13.5)
A007L	Dried pasta	2	14.28	10.0–10.0	338.0-338.0 (338.0)	12.8-12.8 (12.8)	60.0-60.0 (60.0)	2.6-2.6 (2.6)	3.21-3.21 (3.21)	1.0-1.0 (1.0)	0.39-0.39 (0.39)	8.9-8.9 (8.9)
A034X	Hard candies	10	164.60	NA	NA	NA	NA	NA	NA	NA	NA	NA
A0EVD	Isolated proteins and other protein products	4	50.54	20.0–34.0	393.0–527.0 (491.8)	64.7–76.0 (69.1)	2.7–16.4 (7.48)	0.4–6.3 (2.2)	7.1–11.6 (9.58)	1.3–4.5 (3.1)	0.90–2.00 (1.18)	2.7–3.4 (2.9)
A01BN	Peanut butter	1	12.08	NA	NA	NA	NA	NA	NA	NA	NA	NA
A0EYP	Preserved or partly preserved sausages	1	38.78	NA	444.0–444.0 (444.0)	63.9–63.9 (63.9)	4.7-4.7 (4.7)	0.0-0.0 (0.0)	18.3-18.3 (18.3)	5.6-5.6 (5.6)	6.66-6.66 (6.66)	0.1-0.1 (0.1)
A043V	Savory sauces	1	28.95	14.0-14.0	328.0–328.0 (328.0)	6.5-6.5 (6.5)	6.1-6.1 (6.1)	2.9-2.9 (2.9)	31-31 (31)	2.9-2.9 (2.9)	1.300-1.3 (1.30)	NA
A06HL	Snacks other than chips and similar—insect powder	9	120.00	100.0-100.0	259.0–505.0 (427.2)	21.9–74.4 (57.2)	1.0–51.7 (13.2)	0.0–0.8 (0.4)	2.6–26.4 (15.83)	1.7–10.3 (6.52)	0.10–8.10 (2.27)	2.7–9.5 (6.1)
A06HL	Snacks other than chips and similar—whole insect	59	183.62	100.0-100.0	245.0–670.0 (450.8)	14.0–64.8 (43.7)	0.1–39.0 (8.5)	0.0–28 (4.9)	6–49 (20.73)	0–29 (6.98)	0.00–8.8 (1.78)	3.7–6.9 (5.1)
A0EQR	Sweet bars and other formed sweet masses	8	84.64	10.0-10.0	311.0–446.0 (338.0)	13.0–33.0 (29.0)	43.0–46.0 (43.6)	4.9–40.0 (11.9)	9.8–22 (12.24)	2–4 (3.6)	0.09–0.24 (0.21)	4.0–31.0 (25.6)
A03PD	Unsweetened spirits	2	51.62	NA	NA	NA	NA	NA	NA	NA	NA	NA

The average price is expressed in EUR/Kg or L per beverage. Insect %, Kcal, Proteins, Carbohydrates, Sugar, Total fats, Saturated Fatty Acid (SFA), Salt, and Fiber values are reported as minimum—maximum and (average). Nutritional values refer to 100 g. NA: not available information.

**Table 4:** Recommended Dietary Allowances (RDAs) of insects-based food products.

FoodEx2 Code	Food Category	Kcal Per Serving	%RDA Kcal	Proteins per Serving (g)	%RDA Proteins	Carbohydrates per Serving (g)	%RDA Carbohydrates	Sugars per Serving (g)	%RDA Sugars	Total fats per Serving (g)	%RDA Total Fats	SFA per Serving (g)	%RDA SFA
Mealworm (*Tenebrio molitor*)													
A03MA	Beer and beer-like beverage	NA	NA	NA	NA	NA	NA	NA	NA	NA	NA	NA	NA
A009V	Biscuits	434.25	21.71	9.58	12.77	59.75	23.90	22.00	58.67	17.25	22.12	1.23	15.31
A00EY	Cereal bars	465.20	23.26	15.25	20.33	47.10	18.84	27.00	72.00	22.05	28.27	3.65	45.63
A0EQX	Chips/crisps	451.00	22.55	10.98	14.64	58.80	23.52	1.32	3.52	18.32	23.49	2.20	27.50
A0EQD	Chocolate and similar	542.23	27.11	10.90	14.53	40.54	16.22	31.10	82.93	37.21	47.71	17.05	213.13
A005Y	Crackers and breadsticks	463.44	23.17	18.22	24.30	47.13	18.85	2.17	5.78	20.17	25.85	7.89	98.61
A007L	Dried pasta	387.16	19.36	19.24	25.65	57.28	22.91	3.48	9.28	7.58	9.72	1.52	19.00
A034X	Hard candies	234.00	11.70	1.00	1.33	95.00	38.00	0.00	0.00	1.00	1.28	1.00	12.50
A03XF	Meat burger	263.00	13.15	19.70	26.27	1.30	0.52	1.30	3.47	19.40	24.87	6.60	82.50
A03TE	Meat imitates	275.00	13.75	16.60	22.13	19.60	7.84	3.80	10.13	13.50	17.31	2.40	30.00
A00EJ	Muesli and similar mixed breakfast cereals	431.00	21.55	12.00	16.00	51.40	20.56	17.90	47.73	17.70	22.69	7.20	90.00
A0CSK	Pre-mixes (dry) for baked products	546.65	27.33	22.00	29.33	31.60	12.64	0.85	2.27	34.10	43.72	4.53	56.56
A043V	Savory sauces	135.35	6.77	3.75	5.00	7.35	2.94	5.15	13.73	9.25	11.86	1.25	15.63
A06HL	Snacks other than chips and similar—insect powder	507.67	25.38	54.21	72.28	4.08	1.63	0.51	1.36	30.23	38.76	6.01	75.08
A06HL	Snacks other than chips and similar—whole insect	495.16	24.76	48.93	65.24	8.85	3.54	2.04	5.44	28.43	36.45	6.52	81.56
A0EQR	Sweet bars and other formed sweet masses	452.50	22.63	20.00	26.67	36.17	14.47	24.33	64.89	23.50	30.13	3.37	42.08
A00FD	Tortilla chips	244.00	12.20	13.80	18.40	36.20	14.48	2.20	5.87	6.60	8.46	1.60	20.00
A03PD	Unsweetened spirits	NA	NA	NA	NA	NA	NA	NA	NA	NA	NA	NA	NA
House cricket (*Acheta domesticus*)													
A009V	Biscuits	537.00	26.85	15.42	20.56	36.24	14.50	12.26	32.69	35.60	45.64	10.80	135.00
A00EY	Cereal bars	407.33	20.37	14.47	19.29	35.47	14.19	9.37	24.98	20.47	26.24	4.73	59.17
A0EQX	Chips/crisps	375.45	18.77	24.47	32.62	44.92	17.97	4.61	12.30	13.06	16.74	1.86	23.19
A0EQD	Chocolate and similar	522.17	26.11	23.53	31.37	29.04	11.62	24.50	65.33	33.17	42.53	16.73	209.11
A005Y	Crackers and breadsticks	458.19	22.91	22.09	29.45	41.40	16.56	3.40	9.07	21.48	27.53	3.24	40.47
A007L	Dried pasta	345.75	17.29	35.15	46.87	40.80	16.32	1.08	2.87	5.50	7.05	1.70	21.25
A034X	Hard candies	234.00	11.70	1.00	1.33	95.00	38.00	0.00	0.00	1.00	1.28	1.00	12.50
A0EVD	Isolated proteins and other protein products	368.00	18.40	53.00	70.67	18.25	7.30	1.40	3.73	10.60	13.59	6.85	85.63
A03TE	Meat imitates	392.80	19.64	40.34	53.79	4.74	1.90	0.26	0.69	11.40	14.62	6.56	82.00
A00EJ	Muesli and similar mixed breakfast	442.97	22.15	13.73	18.31	49.50	19.80	13.90	37.07	20.17	25.85	3.53	44.17
A0CSK	Pre-mixes (dry) for baked products	336.00	16.80	22.00	29.33	55.00	22.00	0.80	2.13	4.50	5.77	1.50	18.75
A06HL	Snacks other than chips and similar—insect powder	448.00	22.40	67.53	90.03	6.88	2.75	0.13	0.34	17.33	22.22	6.35	79.36
A06HL	Snacks other than chips and similar—whole insect	425.98	21.30	51.27	68.36	8.53	3.41	3.49	9.31	21.35	27.37	7.19	89.91
A0EQR	Sweet bars and other formed sweet masses	424.33	21.22	22.04	29.39	26.78	10.71	20.64	55.05	21.94	28.13	9.41	117.64
A03PD	Unsweetened spirits	NA	NA	NA	NA	NA	NA	NA	NA	NA	NA	NA	NA
Lesser mealworm (*Alphitobius diaperinus*)													
A0EQD	Chocolate and similar	580.00	29.00	8.80	11.73	35.00	14.00	29.00	77.33	43.00	55.13	26.00	325.00
A007L	Dried pasta	373.20	18.66	17.82	23.76	66.22	26.49	2.74	7.31	3.74	4.79	1.02	12.75
A034X	Hard candies	234.00	11.70	1.00	1.33	95.00	38.00	0.00	0.00	1.00	1.28	1.00	12.50
A0EVD	Isolated proteins and other protein products	357.23	17.86	58.49	77.99	5.39	2.15	3.02	8.06	11.84	15.18	4.05	50.56
A03TE	Meat imitates	195.00	9.75	15.60	20.80	5.20	2.08	0.30	0.80	9.70	12.44	6.40	80.00
A00EJ	Muesli and similar mixed breakfast cereals	413.00	20.65	13.68	18.23	52.80	21.12	17.05	45.47	15.15	19.42	2.75	34.38
A01BN	Peanut butter	582.00	29.10	31.46	41.95	8.99	3.60	6.20	16.53	46.84	60.05	6.96	87.00
A00EN	Porridge (dry form—to be diluted)	403.78	20.19	20.87	27.82	43.52	17.41	15.30	40.79	14.12	18.10	5.27	65.88
A0CSK	Pre-mixes (dry) for baked products	356.50	17.83	16.50	22.00	58.00	23.20	16.03	42.73	4.73	6.06	1.00	12.50
A06HL	Snacks other than chips and similar—insect powder	510.50	25.53	60.77	81.02	5.35	2.14	0.50	1.33	27.00	34.62	9.63	120.31
A06HL	Snacks other than chips and similar—whole insect	501.00	25.05	59.25	79.00	3.80	1.52	0.62	1.65	27.06	34.70	9.63	120.42
A0EQR	Sweet bars and other formed sweet masses	408.17	20.41	16.73	22.31	42.63	17.05	37.71	100.56	16.76	21.49	2.98	37.29
Grasshopper (*Locusta migratoria*)													
A0EQD	Chocolate and similar	371.00	18.55	43.00	57.33	21.00	8.40	17.00	45.33	10.00	12.82	4.00	50.00
A034X	Hard candies	NA	NA	NA	NA	NA	NA	NA	NA	NA	NA	NA	NA
A06HL	Snacks other than chips and similar—insect powder	595	29.75	48.2	64.27	1.1	0.44	NA	NA	38.1	48.85	13.1	163.75
A06HL	Snacks other than chips and similar—whole insect	449.16	22.458	45.948	61.264	6.932	2.77	1.96	5.24	24.42	31.31	8.05	100.69
Other species													
A009V	Biscuits	NA	NA	NA	NA	NA	NA	NA	NA	NA	NA	NA	NA
A0EQX	Chips/crisps	434.54	21.73	21.54	28.72	44.84	17.94	3.00	8.00	15.30	19.62	1.60	20.00
A0EQD	Chocolate and similar	503.12	25.16	13.16	17.55	38.08	15.23	35.08	93.55	30.58	39.21	15.46	193.25
A005Y	Crackers and breadsticks	420.33	21.02	24.40	32.53	33.67	13.47	2.13	5.69	19.13	24.53	2.87	35.83
A007L	Dried pasta	338.00	16.90	12.75	17.00	60.00	24.00	2.64	7.04	3.21	4.12	1.00	12.50
A034X	Hard candies	NA	NA	NA	NA	NA	NA	NA	NA	NA	NA	NA	NA
A0EVD	Isolated proteins and other protein products	491.75	24.59	69.08	92.10	7.48	2.99	2.15	5.73	9.58	12.28	3.13	39.06
A01BN	Peanut butter	NA	NA	NA	NA	NA	NA	NA	NA	NA	NA	NA	NA
A0EYP	Preserved or partly preserved sausages	444.00	22.20	63.90	85.20	4.70	1.88	NA	NA	18.30	23.46	5.60	70.00
A043V	Savory sauces	328.00	16.40	6.50	8.67	6.10	2.44	2.90	7.73	31.00	39.74	2.90	36.25
A06HL	Snacks other than chips and similar—insect powder	428.14	21.41	57.14	76.19	13.99	5.59	0.60	1.60	15.47	19.84	6.53	81.63
A06HL	Snacks other than chips and similar—whole insect	434.30	21.72	43.35	57.80	9.32	3.73	7.51	20.04	20.48	26.26	8.14	101.77
A0EQR	Sweet bars and other formed sweet masses	338.00	16.90	29.00	38.67	43.60	17.44	11.92	31.79	12.24	15.69	3.60	45.00
A03PD	Unsweetened spirits	NA	NA	NA	NA	NA	NA	NA	NA	NA	NA	NA	NA

Recommended Dietary Allowances (RDAs): 2000 Kcal/day, 75 g proteins/day, 250 g carbohydrates/day, 37.5 g sugars/day, 78 g total fats/day, 8 g saturated fatty acids/day. Values per serving calculated on 100 g of portion. NA: not available information.

## Data Availability

The original contributions presented in this study are included in the article. Further inquiries can be directed to the corresponding author.
